# Progressive Inflammatory Coupling Drives Glaucoma Progression - a Longitudinal Analysis of Cytokine-RNFL Dynamics

**DOI:** 10.22336/rjo.2025.79

**Published:** 2025

**Authors:** Raluca Neacșa, Daniela Manasia, Cristiana Tănase, Mădălina-Elena Tobă, Adina-Diana Moldovan

**Affiliations:** 1 Department of Medico-Surgical Disciplines, Faculty of Medicine, “Titu Maiorescu” University of Bucharest, Bucharest, Romania; 2 Department of Preclinical Disciplines, Faculty of Medicine, “Titu Maiorescu” University of Bucharest, Bucharest, Romania; 3 Witting Clinical Hospital, Bucharest, Romania; 4 “Victor Babeş” National Institute, Pathology Department, Biochemistry, Proteomics Laboratory, Bucharest, Romania; 5 MedLife SA, Bucharest, Romania

**Keywords:** cytokine, RNFL, untreated POAG, treated POAG, inflammation, RNFL = Retinal Nerve Fiber Layer, POAG = Primary Open-Angle Glaucoma, IL-1β = Interleukin-1 beta, TNF-α = Tumor Necrosis Factor alpha, IL-6 = Interleukin-6, IOP = Intraocular Pressure, OCT = Optical Coherence Tomography, IL-4 = Interleukin-4, IL-10 = Interleukin-10, ANOVA = Analysis of Variance, PGAs = Prostaglandin Analogs, SD = Standard Deviation, RGCs = Retinal Ganglion Cells, IFN-γ = Interferon gamma

## Abstract

**Background:**

Inflammatory biomarkers in glaucoma have shown promise but lack the longitudinal analysis necessary for clinical translation. We developed a comprehensive mixed-effects modeling approach to characterize temporal cytokine-RNFL dynamics and reveal patterns in the cytokine-RNFL progression.

**Methods:**

We analyzed a 24-month longitudinal cohort of 57 patients (19 each: controls, untreated POAG, treated POAG) using: (1) mixed-effects models with individual heterogeneity modeling; (2) censoring-informed cytokine analysis; and (3) temporal correlation network analysis.fi

**Results:**

Mixed-effects models revealed significant group differences in RNFL progression: controls (-0.20 ± 0.10 μm/year), untreated POAG (-1.94 ± 0.54 μm/year), and treated POAG (-1.06 ± 0.49 μm/year). Cytokine censoring patterns provided biological validation—pro-inflammatory cytokines showed 1.37-fold higher detection in untreated POAG versus controls (TNF-alpha: 85.3% vs. 62.1%), independently confirming elevated disease inflammation. Temporal correlation analysis showed progressive inflammatory coupling, with the TNF-alpha of ~ IL6 correlation increasing from r = 0.23 to r = 0.49 over 24 months.

**Discussion:**

Our findings suggest that POAG pathogenesis can be linked to a progressive inflammatory dysregulation, in which other cytokines follow increases in an initial inflammatory cytokine. The doubling of the correlation between TNF alpha and IL6 over time could suggest that the disease progression could be self-amplifying.

**Conclusions:**

This study establishes that glaucoma involves progressive inflammatory coupling, in which initially independent cytokine signals evolve into self-reinforcing mechanisms. The differential censoring patterns provide compelling biological validation. These findings enable precision medicine approaches based on inflammatory phenotypes and support early intervention strategies targeting network establishment.

## Introduction

Glaucoma represents an evolving global health challenge, with recent meta-analyses indicating its prevalence will reach 111.8 million individuals by 2040 [**1**]. A significant barrier to effective management is the high rate of undiagnosed disease, estimated at 90% globally and remaining near 50% even in developed nations [**2**]. This diagnostic gap exists because current clinical tools have fundamental limitations. Optical Coherence Tomography (OCT) has inherent measurement variability of 2-5 μm, whereas functional perimetry deficits often become apparent only after 30-40% of retinal ganglion cells (RGCs) are lost [**3**].

The pathogenesis of primary open-angle glaucoma (POAG) is now understood as an integrated process in which biomechanical stress of the optic nerve head initiates neurodegeneration, which is later amplified by inflammatory cascades [**4**]. This paradigm shift positions inflammation not as a consequence of injury, but as an active driver of pathology. The inflammatory response follows a defined temporal sequence: mechanical and ischemic stress trigger damage-associated molecular patterns (DAMPs), which activate the NLRP3 inflammasome and lead to IL-1beta secretion—the upstream initiator of the cascade. IL-1beta and TNF-alpha act as primary inflammatory signals, inducing downstream mediators like IL-6, while being counterbalanced by regulatory cytokines (IL-10, IL-4). The balance between these pro- and anti-inflammatory signals determines RGC fate and disease progression. The tear film, as a non-invasive source of inflammatory cytokines, is an ideal medium for biomarker discovery [**3,5**]. Tear cytokines reflect the ocular inflammatory milieu and have been shown to correlate with disease severity and risk of progression [**6,7**].

However, the temporal dynamics of these inflammatory networks remain poorly characterized, with most studies being cross-sectional and lacking longitudinal follow-up to assess cytokine dynamics over disease progression [**7**]. Missing longitudinal data has limited the understanding of temporal changes and causality, reducing the clinical applicability of cytokine biomarkers. Multiple methodological challenges that have consistently undermined the reliability of biomarker studies [**8**]: (i) OCT measurements have inherent variability (2-5 μm) that compounds over time, while cytokine measurements are subject to detection limits and assay variability. Traditional analyses treat these noisy measurements as ground truth, potentially masking genuine biological relationships [**3**]. (ii) Multiplex immunoassays frequently yield measurements below detection limits, particularly for anti-inflammatory cytokines. Censoring rates in glaucoma studies range from 20% to 78%, depending on the cytokine and assay platform [**6**]. Most studies exclude censored observations or use ad hoc imputation methods, both of which introduce bias and reduce statistical power. (iii) Glaucoma progression is inherently longitudinal, with individual patients showing heterogeneous patterns of decline. Cross-sectional studies cannot capture these dynamics, while most longitudinal studies use inappropriate statistical methods that fail to account for within-subject correlation and individual heterogeneity [**9**]. (iv) A critical gap exists in understanding whether inflammatory changes precede structural damage or vice versa. This temporal relationship has profound implications for therapeutic targeting but requires sophisticated analytical approaches to establish [**10**].

To address these challenges, we developed and applied a comprehensive longitudinal analytical framework to characterize temporal relationships between cytokines and RNFL in glaucoma. Our approach explicitly addresses the methodological limitations outlined above through mixed-effects modeling to handle repeated measures and individual heterogeneity, appropriate characterization of censored data patterns, and correlation analysis to examine temporal evolution of inflammatory networks; and (4) examination of temporal precedence in cytokine-RNFL relationships [**11**]. As the primary aim, we expected an inverse relationship between the increasing inflammatory cytokines and the RNFL reduction.

## Methods

### Study Design and Cohort

We analyzed data from a 24-month longitudinal study of 57 subjects allocated to three groups (n = 19 each): healthy controls, untreated POAG, and POAG patients treated with prostaglandin analogue. The percent of missing data was 8,42%.

### Statistical analysis

Linear multilevel models were used to examine longitudinal RNFL progression rates, accounting for repeated measures and individual heterogeneity [**12**]. The general model specification included: the time in months since the start of monitoring, the group (control, untreated, treated), the interaction between the latter two, the patient’s age and sex, and the patient’s diabetic and hypertensive status. The multilevel effects structure allows for individual heterogeneity in both baseline RNFL thickness and progression rates, addressing the known variability in glaucoma progression patterns [**13**].

Model selection was performed using likelihood ratio tests, with the final model selected based on an optimal balance of fit and parsimony.

Left-censored cytokine data, resulting from measurements below the limit of detection, were characterized using methods that appropriately account for censoring patterns [**14**]. Rather than excluding censored observations or using ad hoc imputation methods, we characterized censoring patterns as informative biological signals [**6**]. Detection rates were calculated as the proportion of observations above the detection limit, with particular attention to group-specific patterns that might reflect differential inflammatory burden. We analyzed overall and group-specific censoring rates by cytokine (which cytokines were above the detection threshold). Furthermore, we investigated temporal patterns in censoring rates and the correlation between censoring and disease severity.

The temporal evolution of cytokine relationships was assessed using Pearson correlations calculated at each 6-month time point. This approach captures the dynamic nature of inflammatory networks over time, addressing the limitation of static correlation analyses [**4**]. Key inflammatory interactions were prioritized based on known biological pathways: a) pro-inflammatory coupling: IL-1beta~TNF-alpha, IL-1beta~IL-6, TNF-alpha~IL-6, b) anti-inflammatory coordination: IL-4~IL-10, and c) cross-regulatory interactions: pro- vs. anti-inflammatory cytokines. Correlations were computed using only complete observations, and bootstrap confidence intervals were calculated to assess statistical significance [**12**]. The temporal evolution of correlations was visualized and quantified to identify patterns of progressive coupling or decoupling.

Individual progression patterns were characterized using patient effects extracted from the multilevel regression; thus, patients were classified into progression phenotypes based on their individual slopes: slow progressors (rate > 75th percentile, or the least negative), average progressors (rate between 25th and 75th percentile, and fast progressors (rate < 25th percentile, or the most negative). This classification aimed to examine factors associated with different progression patterns and has direct clinical relevance for risk stratification [**15**].

All models were fitted using R version 4.4.1. Statistical significance was assessed at alpha = 0.05, with effect sizes reported alongside significance tests to facilitate clinical interpretation.

## Results

**Fig. 1:**
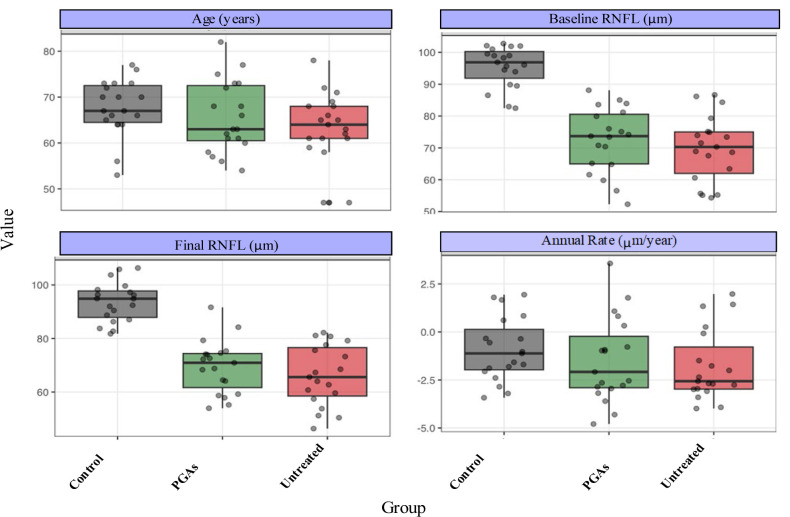
Cohort Characteristics by Group

The cohort consisted of 57 patients equally distributed across three groups. There were 25 missing measurements out of the planned 285. Mean RNFL progression rates were -0.20 ± 0.10 μm/year for controls, -1.94 ± 0.54 μm/year for untreated POAG, and -1.06 ± 0.49 μm/year for treated POAG patients (p < 0.01) (**Fig. 1**).

Cytokine detection rates ranged from 67.0% (IL-10) to 77.5% (IL-1beta), reflecting substantial but manageable levels of censoring, i.e., laboratory values below the detection limit. Group-specific presence or absence of cytokines was congruent with the expected pathophysiological patterns: pro-inflammatory cytokines showed higher detection rates in untreated POAG patients than in controls—TNF-alpha (85.3% vs. 62.1%) and IL-6 (85.3% vs. 69.5%) (**Table 1**).

**Fig. 2:**
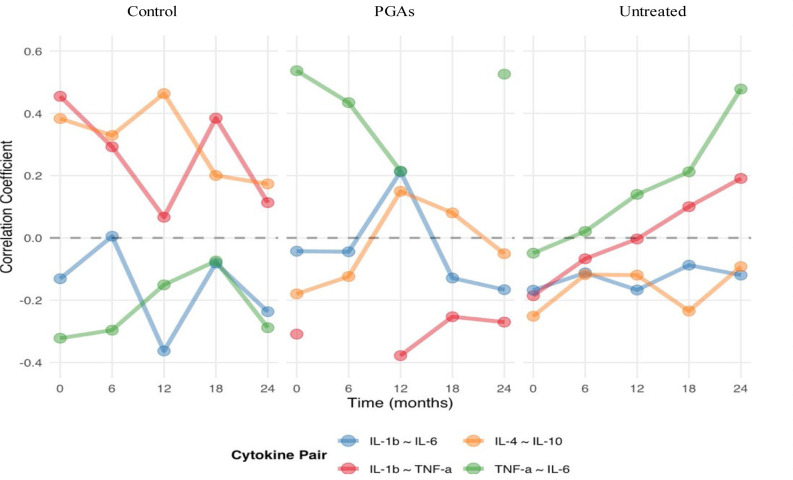
Longitudinal Evolution of Cytokine Correlations

The temporal evolution of cytokine correlations revealed progressive coupling of inflammatory markers over 24 months, providing novel insights into the dynamic nature of inflammatory networks in glaucoma (**Fig. 2**).

The correlation between IL-1beta and TNF-alpha evolved from r = -0.03 (essentially independent) to r = 0.19 (modest positive coupling), suggesting that these pathways become synchronized as disease progresses. This pattern is consistent with the known biological relationship in which IL-1beta can induce TNF-alpha expression, and the temporal evolution suggests that this relationship strengthens over time.

Moreover, the correlation between TNF-alpha and IL-6 increased from r=0.23 to r=0.49, representing a doubling of the strength of the correlation and suggesting the development of feed-forward amplification pathways. Anti-inflammatory cytokines (IL-4, IL-10) exhibited variable correlations, indicating complex regulatory dynamics that may reflect a balance between inflammatory drive and regulatory response.

The individual trajectory analysis revealed substantial heterogeneity within each group, extending beyond the group-level differences. Control patients showed relatively stable RNFL thickness, with minimal decline, whereas POAG patients demonstrated variable progression rates, ranging from slow to rapid decline. Notably, the inflammatory cytokine trajectories exhibited corresponding patterns, with higher baseline levels and steeper increases in faster-progressors.

The multilevel statistical model confirmed significant group differences in RNFL progression rates, providing quantitative validation of the differential progression patterns observed in clinical practice. The interaction terms (Time & Untreated and Time & Treated) were highly significant (p < 0.01), indicating that untreated POAG patients had a significantly faster decline in RNFL than controls.

The random effects structure of the multilevel model revealed substantial individual variability in both baseline RNFL thickness and progression rates, with variance components indicating that about 60% of the total variance in RNFL progression was attributable to personal differences. This implies the existence of multiple (sub-) phenotypes within each group.

The model diagnostic plots confirmed the appropriateness of the mixed-effects modeling approach. Residual patterns showed good model fit with no systematic deviations, and the Q-Q plot indicated approximately normally distributed residuals. The random effects distributions showed the expected patterns, with the negative correlation between random intercepts and slopes indicating that patients with higher baseline RNFL tended to have slower progression rates.

**Table 1:** Cytokine detection rates by group (%)

Group	IL-1b	IL-6	TNF-α	IFN-ᵞ	IL-4	IL-10
Control	87.4	69.5	62.1	65.3	68.4	64.2
PGAs	64.2	72.6	72.6	64.2	71.6	67.4
Untreated	81.1	85.3	85.3	77.9	82.1	69.5
**Overall**	**77.5**	**75.8**	**73.3**	**69.1**	**74.0**	**67.0**

## Discussion

Our longitudinal analysis identified temporal relationships between cytokines and RNFL in POAG and yielded several key findings that explain the inflammatory dynamics underlying POAG progression. By integrating with subsequent longitudinal studies, we hope our work can advance clinical practice and therapeutic development. This study also addressed some fundamental methodological challenges in POAG-biomarker research. The multilevel longitudinal modeling approach appropriately handled the correlation structure inherent in longitudinal data while accommodating individual heterogeneity in progression patterns. The explicit characterization of censoring patterns transformed an analytical challenge into a source of biological insight, demonstrating that apparent “missing data” could contain valuable information about disease state.

Mean RNFL progression rates aligned with established literature values and demonstrated the expected pattern of progression across disease states and treatment

status. Also, the progression rates established the clinical validity of our cohort and provided the foundation for examining how inflammatory biomarkers relate to structural changes over time.

Group-specific presence or absence of cytokines is consistent with the expected pathophysiological patterns: pro-inflammatory cytokines are more frequently detected in untreated POAG patients than in controls, consistent with an elevated inflammatory burden in disease [**6**]. Treated patients showed intermediate detection rates, suggesting partial inflammatory modulation. These detection rates aligned with the inflammatory hypothesis in POAG, showing that censoring should not merely be a technical limitation but also contain essential biological information. The observation that pro-inflammatory cytokines, such as TNF-alpha and IL-6, were detected at higher rates in untreated POAG patients than in controls is consistent with other studies [**8,4**].

The intermediate detection rates in treated patients suggest partial inflammatory modulation, consistent with recent evidence that prostaglandin analogues possess anti-inflammatory properties beyond their IOP-lowering effects [**16**]. This finding had significant implications for treatment selection and monitoring, as it suggested that inflammatory biomarkers could serve as pharmacodynamic markers of treatment response.

The progressive coupling of cytokine correlations over 24 months revealed dynamic inflammatory relationships that would be entirely missed by cross-sectional studies [**7**]. The increase in correlation between IL-1 beta and TNF-alpha could be interpreted as follows: initially, the inflammation pathways were unlinked; concomitant with disease progression, the pathways became more synchronized. This progression pattern indicates that the inflammatory response in POAG is not static but evolves from independent signals to an integrated, amplifying network [**17**]. This possible explanation would also be consistent with our finding that the patients with higher cytokine coupling showed more rapid and consistent RNFL decline.

The increase in the coupling between TNF-alpha and IL-6 over time was even higher, a doubling of the correlation strength over 24 months. Our results suggested the development of feed-forward amplification mechanisms that might explain the progression of POAG, which, when left untreated, often accelerates [**10**]. The strengthening of these inflammatory circuits might represent a critical transition point where the disease becomes self-sustaining, independent of the initial triggering factors.

A POAG treatment and disease monitoring using cytokines could lead to more precision in glaucoma management [**18**]. The progressive coupling patterns, differential detection rates, and individual progression heterogeneity might provide a foundation for patient stratification beyond traditional intraocular pressure. These profiles could guide individualized treatment strategies, with early-stage patients receiving standard IOP-lowering therapy plus inflammatory monitoring, while established inflammatory patients might benefit from combination anti-inflammatory approaches [**3,19**].

The temporal evolution of inflammatory coupling could lead to a novel approach in risk stratification. Patients showing rapid development of cytokine coupling might be identified as high-risk for accelerated progression, warranting more intensive monitoring and potentially more aggressive treatment. Moreover, inflammatory biomarkers could serve as pharmacodynamic endpoints in clinical trials and as monitoring tools in clinical practice.

The progressive coupling patterns might also suggest that the therapeutic window for intervention might be time-dependent. Early intervention targeting the establishment of inflammatory networks might be more effective than treating established circuits. This supports the development of anti-inflammatory adjuvant therapies as early interventions rather than salvage treatments [**11**].

The partial efficacy of prostaglandin analogue treatment, which maintains intermediate inflammatory profiles between controls and untreated patients, could indicate that current therapy incompletely addresses the inflammatory component of glaucoma. As such, we might expect future targeted anti-inflammatory adjuvant therapies. In our interpretation, early-stage interventions could target individual inflammatory pathways before the increase in their correlation, potentially using IL-1beta receptor antagonists or TNF-alpha inhibitors. In later stages of POAG, broad-spectrum anti-inflammatory approaches or combination therapies could be more beneficial.

The biomarker-guided treatment intensification could optimize resource allocation while preventing irreversible vision loss [**20**]. The ability to identify patients at high risk of rapid progression could enable targeted, intensive monitoring, potentially reducing unnecessary visits for low-risk patients while ensuring appropriate care for high-risk individuals. The development of inflammatory biomarkers as companion diagnostics could support more cost-effective healthcare, where treatment decisions are guided by objective biological markers rather than population-based protocols [**21**].

This study had several limitations that should be acknowledged. The cohort size (n=57) limited the power to detect modest effects, and the 24-month follow-up might have been insufficient to capture the whole evolution of inflammatory networks in this slowly progressive disease. The analysis focused on descriptive patterns rather than formal causal inference, and the correlational nature of the findings required validation through mechanistic studies. Future studies should focus on: (i) validating these patterns in larger, more diverse real-world cohorts; (ii) exploring the predictive value of temporal inflammatory patterns for disease progression; (iii) investigating the mechanistic basis of inflammatory coupling; and (iv) developing and testing targeted anti-inflammatory interventions.

## Conclusion

Our longitudinal analysis exposed the temporal dynamics in cytokine activation during POAG progression. The temporal correlation analysis revealed progressive coupling patterns that traditional cross-sectional studies would entirely miss. The evolution of cytokine correlations from independence to coupling over 24 months revealed glaucoma as a disease of progressive inflammatory dysregulation. The transition from discrete inflammatory signals to an integrated, amplifying network suggested that disease progression involved the establishment of self-reinforcing inflammatory circuits that might become independent of initial triggering factors.

The findings had immediate implications for clinical practice and therapeutic development.

The identification of temporal inflammatory phenotypes—early-stage independent signals, established coupling networks, and treatment-responsive profiles—provided a foundation for patient stratification beyond traditional IOP-based approaches. This enabled precision medicine strategies in which treatment decisions are guided by individual inflammatory profiles rather than by population-based protocols.

The partial effectiveness of current prostaglandin analogue therapy in modulating inflammatory markers suggested that inflammatory biomarkers could serve as pharmacodynamic endpoints in clinical trials and as monitoring tools in clinical practice. This could enable more personalized treatment approaches based on individual inflammatory responses.
